# ADHD and gaming addiction in adolescents: psychosocial mediators in the adolescent brain cognitive development study

**DOI:** 10.3389/fpsyt.2026.1756782

**Published:** 2026-03-06

**Authors:** Daniel A. Lopez, Arturo Lopez-Flores, Sara Shao, Bonnie J. Nagel

**Affiliations:** 1 Department of Psychiatry, Oregon Health & Science University, Portland, OR, United States; 2 Center for Mental Health Innovation, Oregon Health & Science University, Portland, OR, United States

**Keywords:** ABCD study, ADHD, adolescents, gaming addiction, psychosocial mediators

## Abstract

**Objective:**

Children with ADHD are at increased risk for developing gaming addiction, but the psychosocial mechanisms underlying this relationship remain poorly understood. This study aimed to identify factors that mediate this risk.

**Method:**

We analyzed data from three consecutive waves (Years 2–4) of the Adolescent Brain Cognitive Development^SM^ (ABCD^®^) Study, including approximately 7,260 participants. Linear mixed-effects models were used to assess the longitudinal association between ADHD diagnosis (measured via the Kiddie Schedule for Affective Disorders and Schizophrenia [KSADS]) and scores on the Video Game Addiction Questionnaire (VGAQ). A parallel mediation model was then employed to evaluate the role of nine psychosocial factors in mediating this relationship.

**Results:**

Children with ADHD had significantly higher VGAQ scores over time, with an average increase of 1.3 points (*p* < 0.001) compared to those without ADHD. The mediation model identified three significant psychosocial mediators: prosocial peer involvement (7.4% of the total effect), school involvement (5.8%), and family conflict (5.1%).

**Conclusion:**

Prosocial peer involvement, school engagement, and family conflict emerged as key psychosocial pathways linking ADHD to gaming addiction. These findings have important public health implications and suggest that strengthening peer networks and family environments may be effective targets for intervention in children with ADHD.

## Introduction

Video games have become a ubiquitous part of childhood and adolescence in the United States, with an estimated 76% of children under the age of 18 regularly engaging in gaming (1). Playing video games offers cognitive benefits, such as enhancing spatial skills, and they also provide opportunities for social interaction (2, 3). However, the widespread use of video games is not without risks. A subset of children may develop problematic gaming habits that can disrupt daily life, impact academic performance, social relationships, and mental health.

Attention-Deficit/Hyperactivity Disorder (ADHD), a neurodevelopmental disorder characterized by inattention, hyperactivity, and impulsivity, may increase susceptibility to gaming addiction (4, 5). Approximately 11.4% of U.S. children aged 3–17 have ever been diagnosed with ADHD, with boys experiencing about double the prevalence compared to girls (6). Research indicates that children with ADHD are significantly more likely to exhibit compulsive video game use, with one study showing a more than three-fold higher rate of problematic gaming compared to their peers without ADHD (7). This heightened vulnerability underscores the need for further research to explore the underlying psychosocial mechanisms driving the association between ADHD and gaming addiction.

Gaming Disorder, often referred to as gaming addiction, is characterized by a pattern of gaming that adversely affects daily functioning, involves preoccupation with gaming and impaired control over gaming frequency and intensity (8). Gaming may also serve as a coping mechanism to escape real-life problems or negative emotions such as anxiety or depression (9). The global prevalence of gaming addiction is estimated at 3%, though it varies based on screening tools and study samples (10). While Internet Gaming Disorder (IGD) and Gaming Disorder are recognized in the DSM-5 and ICD-11, respectively, this study will use the term “gaming addiction” to focus on subclinical symptoms and behaviors rather than official diagnostic criteria to describe clinically diagnosable conditions (5, 11).

The impulsivity and reward-seeking behavior associated with ADHD may contribute to gaming addiction. A longitudinal study with children found that greater impulsivity and ADHD symptom scores were significant risk factors for developing pathological gaming habits (12). Further evidence of the complex relationship between ADHD and gaming addiction comes from clinical intervention studies. For instance, a longitudinal clinical study found that participants with comorbid IGD-ADHD had a 74% lower rate of recovery and a five-fold higher rate of IGD recurrence compared to those with IGD alone (13). Additionally, a five-year treatment study found that higher ADHD symptom severity predicted lower long-term recovery rates from IGD (14). Neurobiologically, individuals with ADHD or a gaming addiction share common brain alterations in regions associated with reward processing, such as the striatum, orbitofrontal cortex, and anterior cingulate cortex (15). These findings suggest that while neurobiological factors contribute significantly to the relationship between ADHD and gaming addiction, understanding the full scope of this connection also requires examining psychosocial factors that may play a critical role.

Psychosocial factors are essential in understanding the development and maintenance of gaming addiction, especially among individuals with ADHD. While previous research has explored the role of family and school relationships in gaming addiction, studies specifically focusing on children with ADHD are limited and have largely relied on cross-sectional designs, often examining individual psychosocial domains in isolation. For instance, problematic gamers have been found to experience more family conflict and lower family cohesion compared to their non-problematic peers (16). However, other studies have reported inconsistent findings, with some not finding a significant association between parent-child relationships and gaming addiction symptoms (17, 18). Similarly, poor academic performance has been linked to an increased risk of gaming addiction, while greater school integration has been associated with lower gaming addiction symptom scores (18, 19). Despite these insights, there remains a clear gap in the literature regarding the longitudinal and multilevel psychosocial pathways through which ADHD contributes to gaming addiction risk.

Children with ADHD experience disproportionate difficulties across multiple social systems, including family regulation, school engagement, and peer relationships, each of which may heighten vulnerability to maladaptive coping behaviors such as excessive gaming (20, 21). Developmental and addiction frameworks suggest that deficits in self-regulation, environmental structure, and social reinforcement increase reliance on highly rewarding, easily accessible behaviors for mood regulation and escape (22, 23). Within this context, elevated family conflict and reduced parental monitoring may limit behavioral regulation at home, school disengagement may increase unsupervised time and stress-related avoidance, and peer contexts may either buffer or amplify problematic gaming depending on the presence of prosocial versus delinquent influences.

### Study aims

In this study, we examined longitudinal associations between ADHD and gaming addiction symptoms in a large, diverse cohort of adolescents enrolled in the Adolescent Brain Cognitive Development^SM^ (ABCD^®^) Study. We also investigated the potential mediating role of psychosocial factors. Prior studies have largely focused on cross-sectional associations or single domains of influence. The present study extends this literature by simultaneously examining multiple psychosocial mediators longitudinally, allowing for a more nuanced understanding of potentially modifiable pathways linking ADHD to gaming addiction symptoms.

Gaming addiction symptoms were measured using the Video Game Addiction Questionnaire (VGAQ) across three time points. ADHD was assessed using the Kiddie Schedule for Affective Disorders and Schizophrenia for School-Aged Children (KSADS-COMP) at the same three time points. The mediators analyzed included a broad range of factors, encompassing family dynamics, school experiences, and peer behaviors. We hypothesized that ADHD diagnosis would predict higher subsequent gaming addiction symptoms and that this association would be partially mediated by greater family conflict, lower parental monitoring, poorer school engagement, and less prosocial peer involvement over time. Specifically, we expected that higher family conflict and school disengagement would be associated with increased gaming addiction symptoms, whereas greater parental monitoring, school involvement, and prosocial peer relationships would be associated with lower symptoms.

## Materials and methods

The ABCD Study is an ongoing longitudinal cohort study that enrolled 11,868 aged 9–10 children (and their parent/guardian) from 21 sites throughout the United States (24). The study includes annual assessments until participants are 19–20 years old. The sample includes more than 1,000 twin pairs and nearly 1,000 sibling pairs, alongside approximately 8,000 singletons. Detailed exclusion criteria are available on the ABCD Data Documentation website (docs.abcdstudy.org) and include severe medical conditions (e.g., severe neurological disorders), contraindications for MRI (e.g., metal implants), non-English speaking, significant sensory impairments (e.g., severe hearing impairment), and an existing diagnosis of schizophrenia, alcohol/substance use disorder, cognitive impairment or intellectual disability, or moderate-to-severe autism spectrum disorder.

Participants, along with their parent/guardian, completed a series of questionnaires at each study visit (25). The questionnaires encompassed a broad range of topics, including engagement with video games and youth-reported psychosocial experiences (e.g., family conflict). Parents provided information on household income, marital status, and other sociodemographic characteristics at each visit.

The ABCD dataset grows and changes over time. The ABCD data used in this report came from https://doi.org/10.82525/jy7n-g441. DOIs can be found at https://www.nbdc-datahub.org/abcd-release-6-0.

### Data collection

The current study utilized data from the 2-, 3-, and 4-year follow-up assessments because symptoms of gaming addiction, our primary outcome measure, were not measured at baseline nor the year-1 visit. The analytic sample included children who 1) completed the Video Game Addiction Questionnaire (VGAQ) and 2) had a summary score on the Attention Deficit/Hyperactivity Disorder (ADHD) module of the parent-completed Kiddie Schedule for Affective Disorders and Schizophrenia for School-Aged Children (KSADS-COMP). Participants with missing data on the mediating variables were excluded from the mediation analysis.

For the longitudinal multilevel models, ADHD diagnosis and VGAQ scores were drawn from repeated assessments at the Year-2, Year-3, and Year-4 follow-up visits. For the parallel mediation analyses, ADHD diagnosis was assessed at Year-2, psychosocial mediators were assessed at Years 2 and 3, and VGAQ outcomes were assessed at Year-4. Because the Children’s Report of Parental Behavior Inventory was not administered at Year-2, baseline adjustment for this mediator utilized the Year-1 assessment.

### Measures

#### Symptoms of gaming addiction

The primary outcome, symptoms of gaming addiction, was measured using the ABCD Video Game Addiction Questionnaire (VGAQ), an adaptation of the Bergen Facebook Addiction Scale (26) (**Supplementary Table 1**). The VGAQ measure has previously been found to have a unidimensional factor structure, along with measurement invariance across age and race/ethnicity and measurement variance by sex (27). The VGAQ was only answered at each study visit by children that reported playing video games (i.e., the measure was skipped for children that reported not playing video games).

VGAQ items were scored on a six-point Likert-type scale (1=Never, 2=Very rarely, 3=Rarely, 4=Sometimes, 5=Often, 6=Very often) (27). Items were recoded as 0=Never to 5=Very Often to improve interpretation of results. The six items of the VGAQ cover the core elements of addiction: salience, mood modification, tolerance, withdrawal symptoms, conflict, and relapse (28). A total VGAQ score was calculated for each participant by summing item responses, with higher scores indicating greater symptoms of gaming addiction. A composite score is recommended when there are five or more response options and strong internal consistency (29, 30). The McDonald’s Omega (ω) of the VGAQ demonstrated good internal consistency: 0.89 at year-2, 0.89 at year-3, and 0.91 at year-4. The possible range of scores was 0 to 30.

#### ADHD diagnosis

ADHD was assessed using the ADHD module of the KSADS-COMP, which includes 93 variables used to create summary scores and diagnostic estimates. The following codes were included in the ADHD KSADS variable: 1=present, 0=absent, 888=Question not asked due to primary question response (branching logic). The 888 responses were recoded as 0=absent since those participants did not meet the requirements for the full ADHD screener (i.e., no symptoms of ADHD). For this study, we focused on the ADHD Present diagnostic summary (variable name: mh_p_ksads:adhd:pres_dx) due to its immediate relevance in gaming addiction. The ADHD Present diagnosis required impairment in functioning in two domains (e.g., social life and school performance). Additional information on the diagnostic criteria can be found on the ABCD Data Documentation page (docs.abcdstudy.org/latest/documentation/non_imaging/mh.html).

### Psychosocial mediators

All psychosocial mediators were youth-reported measures selected based on their theoretical relevance to ADHD and gaming addiction.

#### Parental monitoring

The Parental Monitoring survey is a 5-item subscale measuring the youth’s perception of everyday parental oversight (31) (**Supplementary Table 2**). Items were answered on a five-point scale (1=Never, 2=Almost Never, 3=Sometimes, 4=Often, 5=Always or Almost Always). Higher scores indicate greater parental awareness and effort to shield youth from risky behaviors (31).

#### Family environment scale – family conflict

The Family Conflict subscale of the Family Environment Scale consisted of 9 items on a binary scale (1=Yes, 0=No) assessing the proximal social environment (31–33) (**Supplementary Table 3**). A summary score was calculated by summing the number of endorsed items and dividing by the number of items completed, with higher average scores indicating greater family conflict.

#### School risk and protective factors

The School Risk & Protective Factors (SRPF) scale consisted of 12 items that examined youth’s perceptions of their school climate and school engagement (34, 35) (**Supplementary Table 4**). The ABCD version of the SRPF produced three separate subscale scores: School Environment (6 items), School Disengagement (2 items), and School Involvement (4 items) (31, 33). Items were answered on a four-point scale (1=NO!, 2=no, 3=yes, 4=YES)!, and subscale items were summed to obtain a total subscale score. A higher score on the School Environment subscale denoted greater youth-perceived school opportunities and support. A higher score on the School Involvement subscale was interpreted as greater positive involvement in school. A higher score on the School Disengagement subscale indicated greater alienation from school.

#### Prosocial behavior scale

The Prosocial Behavior questionnaire measured the youth’s tendency to help others (36). The ABCD version included 3 items from the original measure on a three-point scale (0=Not True, 1=Somewhat True, 2=Certainly True) (31) (**Supplementary Table 5**). Higher mean scores were interpreted as having greater prosocial behaviors.

#### Peer behavior profile

The Peer Behavior Profile (PBP) consisted of 6 items that were used to create the PBP Prosocial Peer Involvement subscale and the PBP Rule Breaking/Delinquent Peer Involvement subscale (31, 37) (**Supplementary Table 6**). The PBP Prosocial Peer Involvement subscale measured the youth’s engagement with peers that are athletes, attend church, or are excellent students. The Rule Breaking/Delinquent Peer Involvement subscale measured the youth’s engagement with peers that skip school, have been suspended, or shoplift occasionally. The items were on a five-point scale (1=None or almost none, 2=A few, 3=Half, 4=Most, 5=Almost or almost all). Scores for each subscale were calculated by summing individual items. Higher scores on the PBP Prosocial Peer Involvement subscale were interpreted as having more peers that excel academically and socially. Higher scores on the Rule Breaking/Delinquent Peer Involvement subscale were interpreted as having more antisocial peers.

#### CRPBI acceptance scale

The Children’s Report of Parental Behavior Inventory (CRPBI) Acceptance Scale is a 10-item subscale of primary caregiver warmth, acceptance, and responsiveness answered on a three-point scale (1=Not like him/her, 2= Somewhat like him/her, 3= A lot like him/her (38). The ABCD version of the CRPBI included the 5 items with the highest factor loadings (31) (**Supplementary Table 7**). The shortened ABCD version was completed at the year-3 and year-4, but not year-2, study visit. Consequently, while baseline adjustment for other mediators in the model used Year-2 values, baseline adjustment for the CRPBI specifically used the Year-1 score (since the CRPBI was not administered at Year-2). Higher mean scores on the CRPBI indicated greater youth-perception of primary caregiver warmth, acceptance, and responsiveness.

### Statistical analysis

#### Longitudinal multilevel model

A multilevel linear regression model was used to examine the association between ADHD diagnosis and VGAQ score over time. The multilevel model accounted for the nesting of individuals within families by using a random intercept. A random slope was included for the visit number (year-2 = 1, year-3 = 2, year-4 = 3). Covariates included sex, age, household income, household education, marital status, study site, and neighborhood safety. Neighborhood safety was measured using the Neighborhood Safety/Crime Survey, which consisted of three items on a five-point Likert scale used to create a summary score with higher scores indicating greater neighborhood safety (39).

The beta coefficient and 95% confidence intervals (CI) were reported, with the beta coefficient representing the mean difference in VGAQ score over time in participants with and without an ADHD diagnosis. The model was fit using the *glmmTMB* package in R version 4.3.2 and R Studio version 2023.12.1.402 (40–42). Estimated marginal means were generated using the *emmeans* package in R (43).

#### Parallel mediation model

**Figure 1:**
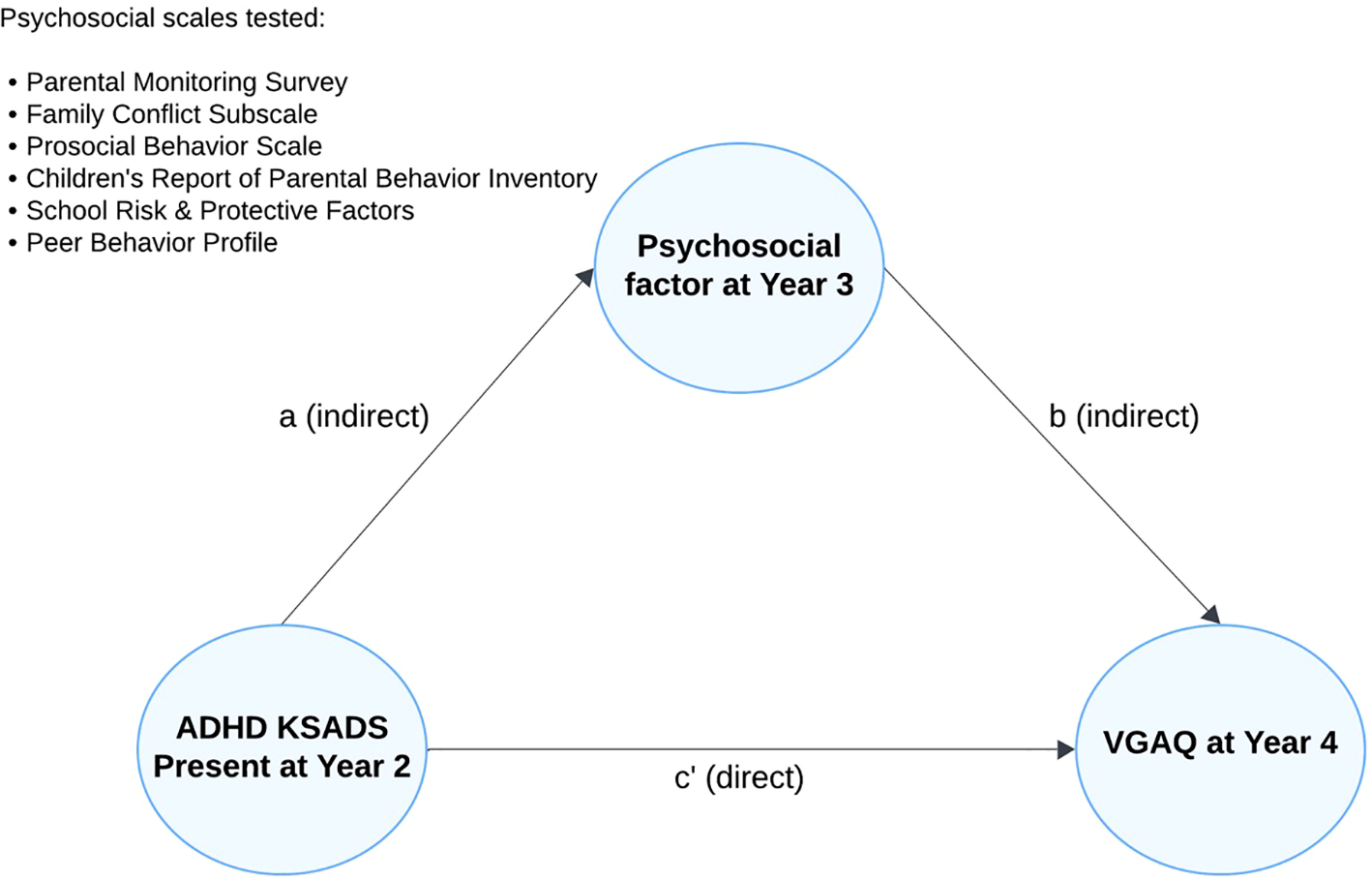
Parallel mediation model testing whether year-3 psychosocial factors mediate the association between year-2 ADHD (KSADS) diagnosis and year-4 Video Game Addiction Questionnaire (VGAQ) scores; arrows depict indirect (a, b) and direct (c′) paths.

A parallel mediation model was conducted to explore the potential psychosocial mechanisms through which ADHD influences gaming addiction symptoms. The analysis utilized multilevel linear regression models in Mplus version 8.10 (44), allowing for the examination of multiple mediators simultaneously, thereby isolating specific pathways and reducing potential biases (45). The model assessed whether ADHD diagnosis at year-2 was associated with psychosocial mediators at year-3, and, in turn, whether these mediators at year-3 predicted VGAQ scores at year-4 (**Figure 1**). Additional details of the parallel mediation model can be found in the **Supplementary Materials**.

Data for the mediation analysis was prepared in R Studio, and included converting the dataset from long to wide format prior to the mediation analysis (41, 42). The wide data was then converted into a data file for Mplus using the *MplusAutomation* package (46). The outcome and mediator models adjusted for sex, age, study site, household income, household education, parent marital status, and neighborhood safety. We included clustering by family ID (TYPE=COMPLEX) to account for nonindependence in observations (i.e., family members are more like each other). Year-2 VGAQ and mediator values were residualized in the outcome model to reduce unmeasured confounding (47). Residual correlations were specified among all mediators to account for shared variance and improve model stability. The beta coefficients and proportion mediated (indirect effect/total effect) were reported, with 95% CIs estimated from 10,000 bootstrapped samples. Estimation was conducted using the Monte Carlo integration algorithm. Given that all indirect effects were estimated simultaneously in a single model with correlated mediators, correction for multiple comparisons was not applied.

Model fit was evaluated descriptively using standard global fit indices available in Mplus. For models estimated without clustering, RMSEA, CFI, and TLI were examined. For the final clustered model (TYPE=COMPLEX), SRMR was used, as other global fit indices are not provided under this specification. Consistent with recommendations for complex longitudinal mediation models, fit indices were interpreted descriptively rather than as strict cutoff-based criteria, in conjunction with theoretical plausibility and stability of parameter estimates. Replication code is available at: https://github.com/Daniel-Adan-Lopez/ABCD_Gaming_ADHD.

#### Missing data

The amount of missing data in the analytic sample was less than 2% for the covariates included in the multilevel model (**Supplementary Table 8**). ADHD diagnosis data were missing for 6.03% of participants across the three study visits. Most of this missingness occurred at year 4 (accounting for 74% of the missing ADHD data), likely due to pandemic-related restrictions on KSADS administration. The VGAQ score was missing from 21.9% of participants, although this was due to missingness by design (i.e., children that reported not playing video games skipped the VGAQ). The amount of missing mediator data ranged from 0.2% to 2.3%.

## Results

### Sample characteristics

The sample was approximately 60% male (**Table 1**). The mean VGAQ score (range = 0–30) across the three time points was 6.4 at year-2, 7.1 at year-3, and 6.4 at the year-4 visit, indicating relatively low scores on average. The proportion of children with a KSADS diagnosis of ADHD was 5%-6% at each study visit. Higher VGAQ scores were observed in males, children with an ADHD diagnosis, children from households with less than a bachelor’s degree, and children from lower income households (**Table 2**).

**Table 1:** Characteristics of participants included in the analytic model.

Characteristic	Year-2 visit (n=7933)	Year-3 visit (n=8100)	Year-4 visit (n=6602)
**Age in years, mean (SD)**	12.1 (0.7)	12.9 (0.65)	14.1 (0.7)
Sex at birth			
Male	4815 (60.7)	4905 (60.6)	4082 (61.8)
Female	3118 (39.3)	3195 (39.4)	2520 (38.2)
Household income			
<$50k	1891 (23.8)	1740 (21.5)	1287 (19.5)
≥$50K to $100K	1981 (25.0)	1991 (24.6)	1466 (22.2)
>$100K	3415 (43.0)	3720 (45.9)	3310 (50.1)
Do not know	322 (4.1)	395 (4.9)	313 (4.7)
Refuse to answer	324 (4.1)	254 (3.1)	226 (3.4)
Household education			
< HS diploma	361 (4.6)	352 (4.3)	266 (4.0)
HS diploma/GED	778 (9.8)	703 (8.7)	585 (8.9)
Some college	2603 (32.8)	2567 (31.7)	2015 (30.5)
Bachelor’s degree	1678 (21.2)	1730 (21.4)	1451 (22.0)
Post graduate degree	2513 (31.7)	2748 (33.9)	2285 (34.6)
Race/ethnicity			
Hispanic	1620 (20.4)	1636 (20.2)	1321 (20.0)
White	4141 (52.2)	4380 (54.1)	3570 (54.1)
Black	1217 (15.3)	1101 (13.6)	937 (14.2)
Asian	141 (1.8)	148 (1.8)	123 (1.9)
Other	814 (10.3)	835 (10.3)	651 (9.9)
Marital status of parent/guardian			
Married or living with partner	5840 (73.6)	6112 (75.5)	4962 (75.2)
Not married	2093 (26.4)	1988 (24.5)	1640 (24.8)
ADHD present			
Yes	469 (5.9)	486 (6.0)	369 (5.6)
No	7464 (94.1)	7614 (94.0)	6233 (94.4)
**VGAQ score, mean (SD)**	6.43 (6.3)	7.09 (6.4)	6.41 (6.2)

SD, Standard deviation; VGAQ, Video Game Addiction Questionnaire.

**Table 2:** Mean VGAQ score across participant characteristics.

Participant characteristic	VGAQ score
	Mean(SD)
Sex	
Male	8.4 (6.3)
Female	4.2 (5.4)
Household income	
<$50k	8.0 (7.0)
≥$50K & $100K	6.8 (6.3)
>$100K	6.0 (5.7)
DNK	7.4 (6.9)
Refuse to answer	7.4 (6.8)
Household education	
< HS diploma	7.8 (7.1)
HS Diploma/GED post	8.0 (7.1)
Some college	7.2 (6.6)
Bachelor	6.5 (6.1)
Post graduate degree	6.0 (5.7)
Race/ethnicity	
Hispanic	7.5 (6.6)
White	6.1 (5.8)
Black	7.8 (7.2)
Asian	7.2 (6.1)
Other	7.0 (6.5)
Marital status of parent/guardian	
Married or living with partner	6.5 (6.1)
Not married	7.6 (6.9)
ADHD present	
Yes	9.0 (6.9)
No	6.6 (6.2)

VGAQ, Video Game Addiction Questionnaire.

### Missingness analysis

Participants with missing mediator data (n = 1,918) differed from those with complete mediator data (n = 9,604) (**Supplementary Table 9**). The missing-mediator group was more likely to be male (54.4% vs 51.9%, p = 0.048), to have a higher prevalence of ADHD (9.7% vs 6.8%, p <.001), and to come from households with lower income and lower caregiver education (both p <.001). They were also less likely to have married caregivers at baseline (52.4% vs 70.9%, p <.001). Participants with missing mediator data lived in less safe neighborhoods on average (lower neighborhood safety scores; p <.001). Age did not differ meaningfully between groups (p = 0.063). These patterns suggest mediator missingness was associated with socioeconomic and contextual risk factors.

### The association between an ADHD diagnosis and VGAQ scores

The final multilevel linear model included approximately 22,687 observations across the three study visits (year-2 = 7933; year-3 = 8100; year-4 = 6602). The model found a significant association between an ADHD diagnosis and symptoms of gaming addiction over time (**Table 3**). On average, children with an ADHD diagnosis had a 1.3-point greater (95% CI: 0.97, 1.61; *p* < 0.0001) VGAQ score over time, after adjustment for covariates. The estimated marginal means were 8.2 (95% CI: 7.7, 8.6) for children with ADHD and 6.9 (95% CI: 6.5, 7.2) for those without ADHD. In other words, we expect the average participant with and without ADHD to have a VGAQ score of 8.2 and 6.9, after controlling for other variables in the model.

**Table 3:** The longitudinal association between KSADS ADHD diagnosis and VGAQ score.

ADHD diagnosis	Video gaming addiction score	*p*
	β (95% CI)	
**ADHD present (ref=no)**		<0.0001
Yes	1.29 (0.97, 1.61)	

Linear models with adjustment for age, sex at birth, household income, parent education, neighborhood crime, parent marital status, and study visit.

Although the large sample size affords high statistical power, the observed effect sizes, particularly the approximately 1.3-point difference in VGAQ scores, represent modest but meaningful differences in gaming addiction symptoms at the population level.

### Parallel mediation model

The model fit indices indicated an overall good fit (RMSEA = 0.047, CFI = 0.946, SRMR = 0.073) (**Supplementary Table 10**). However, the TLI value of 0.85 suggested a poorer fit. It is important to note that the TLI is known to be sensitive to model complexity, particularly when there are many parameters or multiple paths involved, potentially indicating a poorer fit even if the model is well-specified (48).

The mediators were weakly to moderately correlated with each other (**Supplementary Figure 1**). The weak to moderate correlations between the mediators are favorable in the context of parallel mediation analysis. High correlations among mediators could indicate multicollinearity, which can complicate the interpretation of the mediation effects and lead to instability in the estimates.

### Indirect effects between ADHD diagnosis and mediators

ADHD diagnosis at year 2 predicted lower levels of prosocial peer involvement, parental monitoring, youth prosocial behavior, school environment support, school involvement, and caregiver warmth at year 3 (**Supplementary Table 11**). ADHD also predicted higher levels of family conflict, school disengagement, and delinquent peer involvement.

### Indirect effects between mediators and VGAQ scores

**Figure 2:**
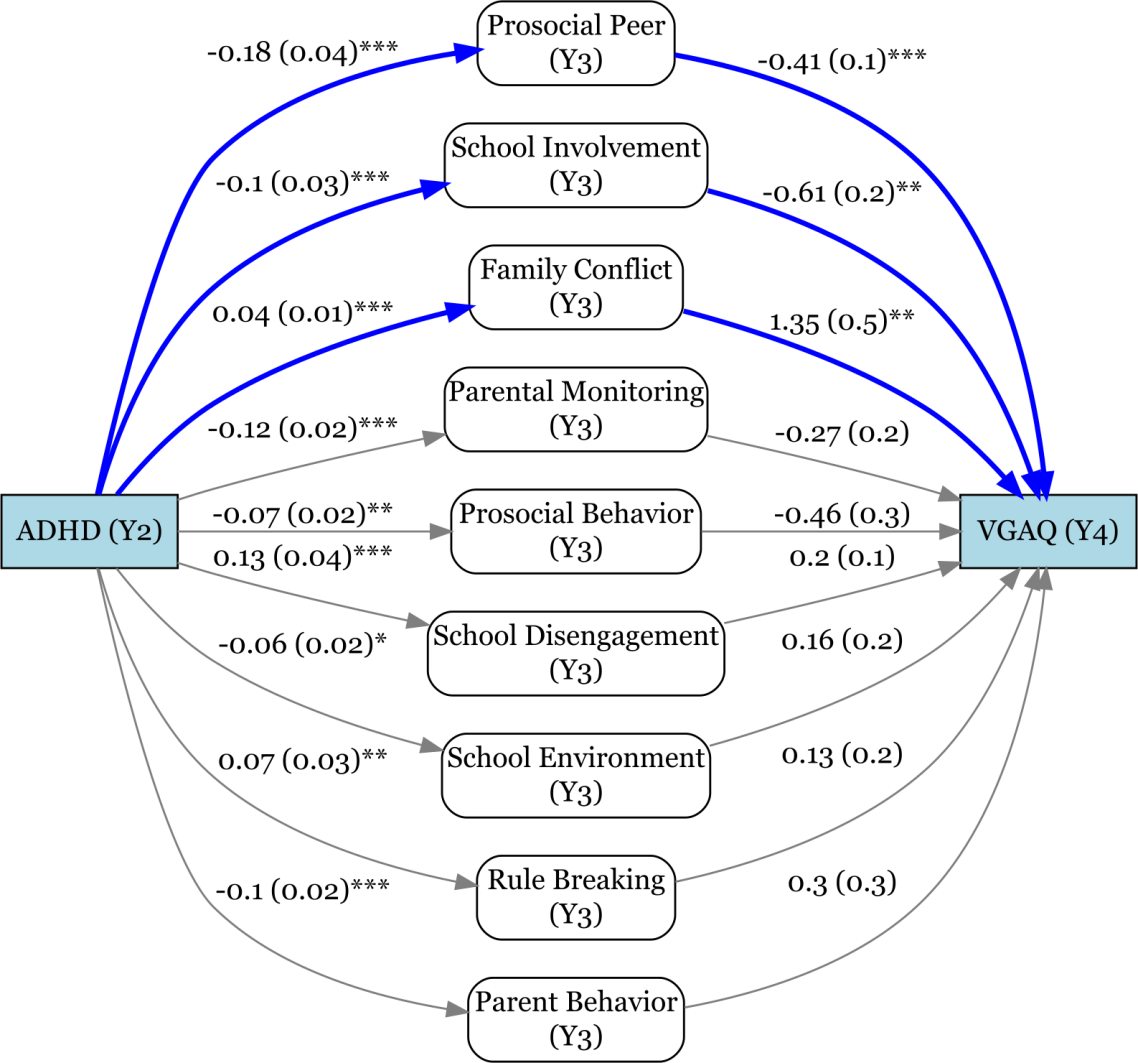
Parallel mediation model showing the indirect effects of child experiences on the association between ADHD status and scores on the Video Game Addiction Questionnaire (VGAQ). ***p <0.001, **p < 0.01, *p < 0.05.

Higher family conflict at year 3 predicted higher VGAQ scores at year 4 (*p* = 0.003; see **Figure 2**), whereas greater school involvement and higher prosocial peer involvement predicted lower VGAQ scores (*p* = 0.005 and *p* < 0.001, respectively).

No other year 3 mediator was significantly associated with VGAQ scores at year 4.

### Mediators in the relationship between ADHD diagnosis and VGAQ scores

The nine mediators collectively accounted for 24.7% of the total effect of an ADHD diagnosis on VGAQ score. Within the parallel mediation model, three mediators emerged as significant contributors: the PBP Prosocial Peer Involvement subscale, the Family Conflict scale, and the SRPF School Involvement subscale. Their respective indirect effects explained 7.4% (*p* = 0.005), 5.1% (*p* = 0.020), and 5.8% (*p* = 0.029) of the total effect (see **Table 4**).

**Table 4:** Indirect effects mediating the relationship between ADHD and symptoms of gaming addiction.

	Indirect effect β (SE)	*p*	Proportion mediated (95% CI)
Mediator			
Parental Monitoring	0.03 (0.03)	0.21	3.3% (-1.8%, 13.3%)
Family Conflict	**0.05 (0.02)**	**0.02**	**5.1% (1.3%, 15.9%)**
SRPF - School Disengagement	0.03 (0.02)	0.2	2.6% (-1%, 11.2%)
SRPF - School Involvement	**0.06 (0.03)**	**0.03**	**5.8% (1.3%, 15.9%)**
SRPF - School Environment	-0.01 (0.02)	0.54	-0.9% (-5.9%, 2%)
Prosocial Behavior Scale	0.03 (0.02)	0.13	3.1% (-0.4%, 11.8%)
PBP Prosocial Peer Involvement	**0.07 (0.03)**	**0.005**	**7.4% (2.6%, 22.7%)**
PBP Rule Breaking/Delinquent Peer Involvement	0.01 (0.02)	0.57	0.9% (-2%, 6.2%)
Child’s Report - Parental Behavior Inventory	-0.03 (0.03)	0.33	-2.5% (-12.3%, 2.8%)

SRPF, School Risk & Protective Factors; PBP, Peer Behavior Profile.

Bold numeric values indicate statistically significant indirect effects (p < 0.05).

No other mediators accounted for a statistically significant proportion of the relationship between ADHD diagnosis and VGAQ scores.

## Discussion

This study underscores the critical role of ADHD in increasing the risk of gaming addiction in adolescents. The longitudinal model indicated that adolescents with an ADHD diagnosis had around a 1-to-2-point higher VGAQ score over time, even after adjusting for potential confounders. The parallel mediation model further identified significant indirect pathways through having prosocial peers, family conflict and school involvement, suggesting these factors help explain how ADHD contributes to an elevated risk of developing gaming addiction.

Path analysis showed that ADHD diagnosis was significantly associated with greater family conflict, which in turn was linked to higher VGAQ scores. ADHD was also associated with lower prosocial peer involvement and reduced school engagement, both of which were in turn associated with higher VGAQ scores.

Although peer, school, and family factors are broadly relevant to adolescent development, our findings demonstrate that these domains do not contribute equally to the ADHD–gaming addiction relationship. Several hypothesized mediators showed minimal or non-significant indirect effects when modeled simultaneously, while others emerged as more prominent pathways. The absence of statistically significant indirect effects does not indicate that these domains are unimportant in shaping youth outcomes. Rather, when modeled simultaneously and accounting for shared variance among psychosocial systems, their unique contributions to gaming addiction symptoms were not detectable within this longitudinal framework. This pattern may reflect overlapping mechanisms across family, school, and peer contexts, modest individual effect sizes, or context-specific influences that were not fully captured in the present design. This differentiation helps refine mechanistic models and suggests that some psychosocial domains may represent more promising targets for intervention than others.

### The association between ADHD diagnosis and symptoms of gaming addiction

Our conclusion that an ADHD diagnosis is significantly associated with greater symptoms of gaming addiction over time aligns with previous research. For example, a meta-analysis of studies exploring the link between symptoms of ADHD and gaming disorder found a moderate relationship, although it noted significant heterogeneity across studies (49). Similarly, Başgül et al. (50) reported significantly higher scores on a computer game addiction scale in adolescents with ADHD compared to controls. Gentile (51) found that 25.3% of children with ADHD met criteria for pathological gaming, compared to 11% of nonpathological gamers. Berloffa et al. (52) also reported a two-fold higher rate of clinically significant gaming addiction symptoms in adolescents with ADHD.

In contrast, our study found an approximate 19% greater average score on the VGAQ in children with an ADHD diagnosis. While the effect sizes observed in this study are smaller than those reported in previous research, they are nonetheless meaningful given the widespread prevalence of gaming and the potential to inform public health strategies.

### Psychosocial mediators involved in gaming addiction risk

Our study reported significant mediation effects by psychosocial factors related to prosocial peer involvement, family conflict, and school involvement, which together accounted for more than 18% of the effect between ADHD diagnosis and gaming addiction symptoms. While literature on psychosocial mediators in this context is limited and often focuses on psychological problems like anxiety and depression, our findings contribute to understanding the broader social factors at play.

### Prosocial peer involvement

The Prosocial Peer Involvement subscale reflects a child’s engagement with peers who participate in structured, socially valued activities such as playing sports, attending church, or performing well academically. While limited research has directly examined the relationship between these peer characteristics and gaming addiction, children with ADHD are consistently found to experience greater social and academic challenges compared to their non-ADHD peers (53). For example, one study reported that children with ADHD experienced significantly lower satisfaction with their peer networks than comparison children (54). In a school-based study, 52% of children with ADHD were rated as peer-rejected, compared to only 14% of comparison children (55). Interestingly, popular peers were more likely to report disliking children with ADHD.

Emerging evidence suggests that a poorly functioning peer network is associated with increased risk for gaming-related problems. In a longitudinal study of adolescents, peer rejection at Time 1 significantly predicted a higher frequency of Internet Gaming Disorder symptoms at Time 3 (56). Another study found that children experiencing greater peer rejection over time showed significantly higher gaming addiction scores than those who maintained consistently low levels of peer rejection (57). Collectively, these findings suggest that children with ADHD may have difficulty forming friendships with peers who exhibit strong prosocial behaviors, potentially increasing their reliance on video games as an alternative social outlet.

While prosocial peer involvement emerged as a significant mediator, delinquent peer involvement did not show a statistically significant indirect effect in the parallel mediation model. This should not be interpreted as evidence that exposure to antisocial peers is unrelated to gaming-related problems. Rather, it suggests that, within this cohort and time frame, the protective influence of engagement with structured, achievement-oriented peer groups may have been more salient than risk conveyed through delinquent peer contexts once correlated psychosocial factors were considered.

### Family conflict

Familial factors are critical risk factors for gaming addiction, especially in children with ADHD. For example, a study of adolescents with IGD and healthy controls reported significantly higher ADHD symptom severity and lower family relationship scores in the former group (58). Lee et al. (13) found a significant inverse relationship between family environment and gaming addiction symptoms in a longitudinal study, highlighting the importance of family environment as a protective factor. Similarly, a longitudinal study involving 3034 children and adolescents found that a warm family environment was a protective factor for later pathological gaming, although their study did not differentiate ADHD symptomatology (59).

Overall, our findings underscore the importance of family-based interventions in mitigating gaming addiction risk, particularly for children with ADHD. Strengthening family relationships may serve as a protective factor against the development of gaming addiction.

Similarly, parental monitoring and caregiver warmth were associated with ADHD diagnosis but did not independently mediate gaming addiction symptoms. These findings may indicate that broader relational climate and family stress processes, rather than discrete parenting behaviors alone, represent more proximal pathways linking ADHD to problematic gaming. Alternatively, these constructs may exert influence through indirect or interactive mechanisms not fully captured in the present model.

### School involvement

The School Involvement subscale, which measures participants’ sense of belonging at school, emerged as another critical factor. The importance of school connectedness has been previously observed in children with symptoms of gaming addiction. For instance, a study involving 833 adolescents reported that school connectedness fully mediated the relationship between parent-adolescent relationship and gaming addiction (60). Rehbein and Baier (18) found that social integration in school reduced susceptibility to gaming addiction in adolescence, while Wei et al. (61) reported that school connectedness partially mediated the relationship between stressful life experiences and online gaming addiction.

Given the significant role of school involvement in reducing gaming addiction risk, schools may be a crucial setting for interventions. Programs that promote school connectedness and engagement could be particularly effective for children with ADHD, potentially reducing their reliance on gaming as a coping mechanism.

### Strengths

The current study had several strengths, First, we utilized a substantial and diverse sample of adolescents from across the United States, enhancing the generalizability of our findings. Second, the analysis included multiple time points of data on ADHD diagnosis, psychosocial mediators, and symptoms of gaming addiction. This longitudinal approach enabled a nuanced understanding of how these variables changed throughout this developmental period and provided a holistic view of the factors influencing gaming addiction. Third, we employed rigorous statistical methods, including adjustment for potential confounding variables and an analysis that accounted for the relationships between mediators. This approach allowed us to identify significant psychosocial factors, such as family conflict and school involvement, as critical targets for intervention. We also minimized the potential for reverse causation by using a lagged mediation analysis, which enabled us to assess the temporal sequencing and mediation effects across three time points. Finally, we used a continuous measure of gaming addiction rather than an arbitrary threshold for disordered gaming. This approach enhanced the precision of our analysis and improved the generalizability of our findings.

### Limitations

Our study has several limitations that should be considered. First, the ADHD diagnosis was based on parent-reported ADHD symptoms using the KSADS-COMP rather than a clinical diagnosis. While the KSADS-COMP is a validated tool for ADHD evaluation, discrepancies may exist when compared to a comprehensive clinical assessment. Second, we did not include a clinical evaluation for diagnosing symptoms of gaming addiction. This limitation may have led to non-differential misclassification of the outcome, assuming that children with and without ADHD did not systematically over- or under-report gaming addiction symptoms. Such misclassification generally biases the results towards the null, potentially underestimating the true association (62). Third, because mediator missingness was more common among participants with lower socioeconomic status, higher neighborhood risk, and ADHD, findings may underrepresent higher-risk youth. Finally, a portion of the data collection coincided with the COVID-19 lockdown, which may have influenced mediator values and the collection of the KSADS ADHD evaluation. Emerging research indicates that adolescents experienced increases in screen time and gaming engagement during pandemic-related lockdowns, alongside declines in school connectedness and mental well-being (63–65). These contextual shifts may have amplified the salience of the mediators identified in the present study, particularly school involvement and family conflict, as traditional sources of structure and social engagement were disrupted. Further research with additional time points is necessary to fully understand the impact of the COVID-19 lockdown on our study findings.

### Implications of the findings

Our findings illuminate the psychosocial mechanisms underlying the relationship between ADHD and gaming addiction symptoms, addressing a critical gap in understanding effective treatment strategies for gaming problems in children and adolescents with ADHD. The results suggest that family-based interventions may benefit from prioritizing reductions in family conflict and strengthening school connectedness, rather than focusing exclusively on discrete parenting behaviors such as monitoring. Interventions that target constructive conflict resolution, consistent routines, and collaborative rule-setting around media use may be particularly effective in mitigating gaming addiction risk.

Notably, psychosocial mediators accounted for only about 25% of the association between ADHD and gaming addiction symptoms, indicating that additional pathways likely contribute to this relationship. Other potentially modifiable factors, such as engagement in structured activities like sports, which have been consistently linked to improvements in cognitive control and self-regulation among children with ADHD (66), warrant further investigation in future research.

Clinically, our findings advocate for a multi-faceted approach to treating gaming problems in children with ADHD that includes targeting improvements in family dynamics and school engagement. From a public health perspective, the findings underscore the potential for school-based interventions aimed at enhancing school connectedness, offering a valuable strategy for supporting children with ADHD in managing gaming behaviors.

## Conclusion

Overall, this study contributes to the growing body of literature on the psychosocial mechanisms underlying gaming addiction, particularly among vulnerable populations such as children and adolescents with ADHD. These insights have important implications for developing targeted interventions aimed at reducing the prevalence of gaming addiction and improving outcomes for at-risk youth.

## Data Availability

The data analyzed in this study is subject to the following licenses/restrictions: The ABCD Study^®^ data used in this article are subject to the terms of the ABCD Data Use Certificate (DUC). Investigators must obtain a DUC and agree to all associated restrictions, including maintaining data confidentiality, prohibiting attempts to re-identify participants, using the data solely for approved research purposes, and complying with all data-security and reporting requirements. Access to the data is available upon application through the NIH Brain Development Cohorts Data Sharing Platform.” Requests to access these datasets should be directed to https://doi.org/10.82525/jy7n-g441.
