# Supplementary material for: ADHD and gaming addiction in adolescents: psychosocial mediators in the adolescent brain cognitive development study
Source: Front Psychiatry. 2026 Mar 6;17:1756782. doi: 10.3389/fpsyt.2026.1756782 (PMC13002843; doi:10.3389/fpsyt.2026.1756782)
Supplement: Supplementary file 1 [file Supplementaryfile1.docx]

**Supplementary Materials**

**Table S1. Video Game Addiction Questionnaire**

**
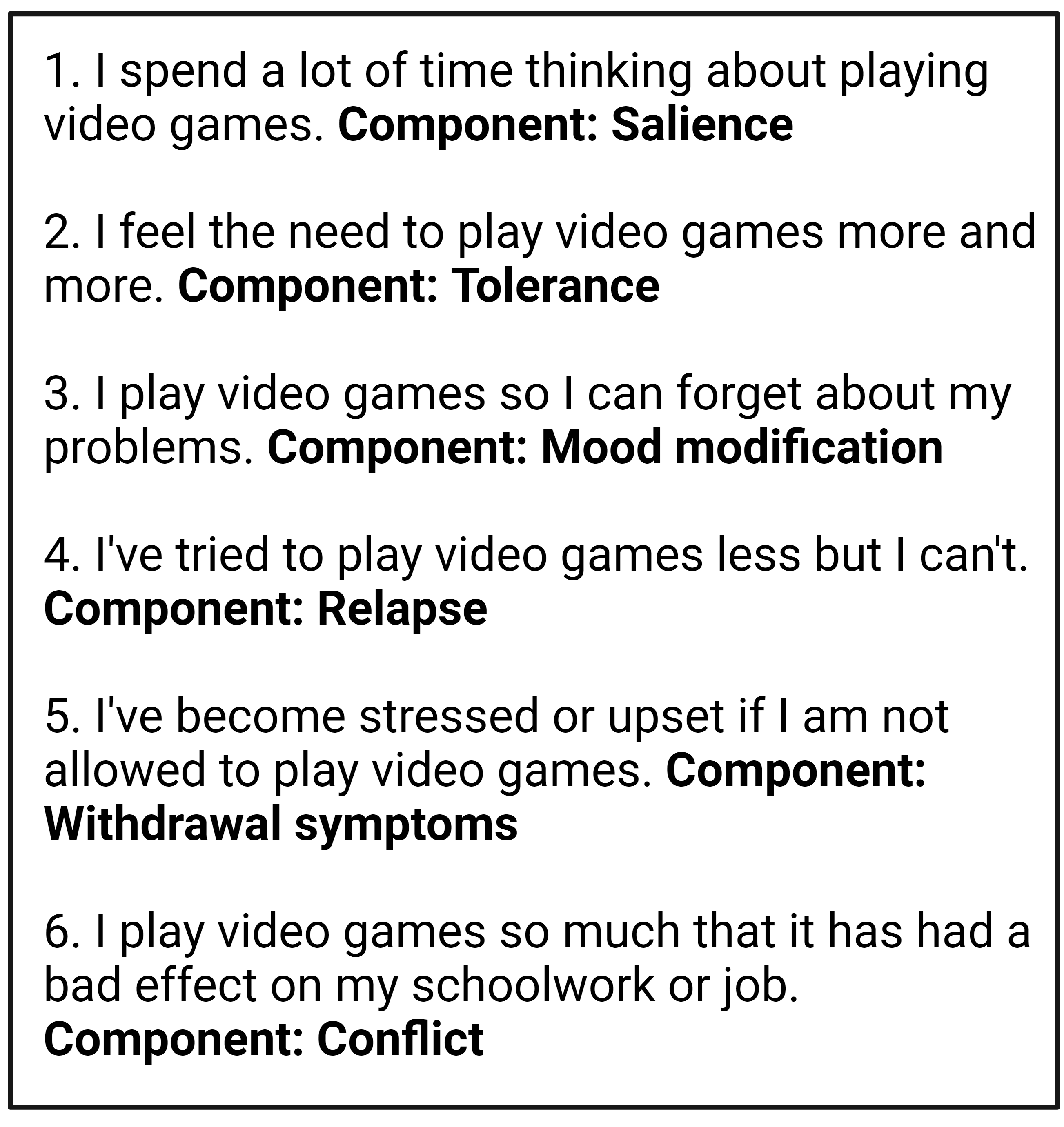
**

**Table S2. Parental Monitoring Survey**

**
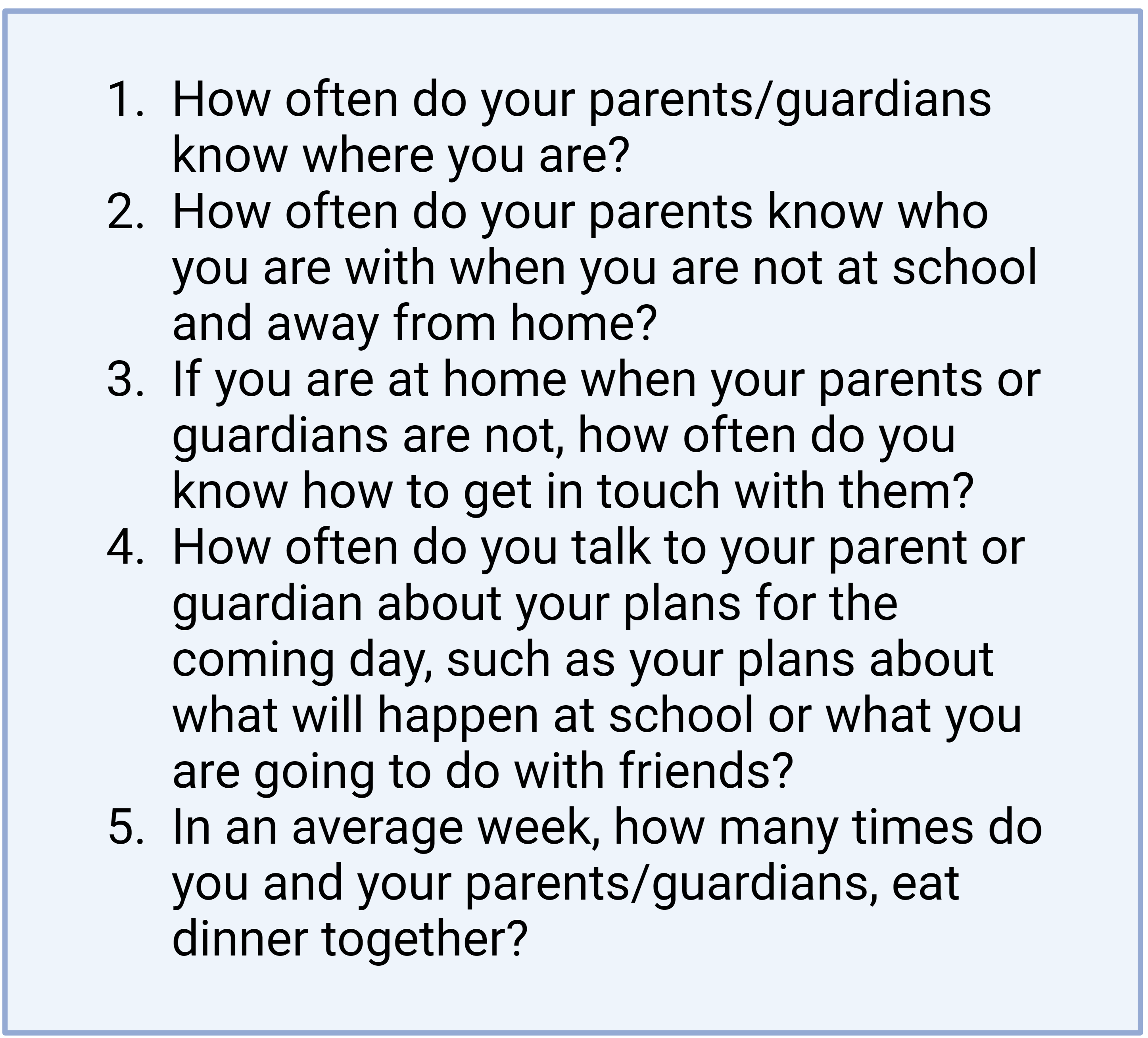
**

**Table S3. Family Environment - Family Conflict subscale**

**
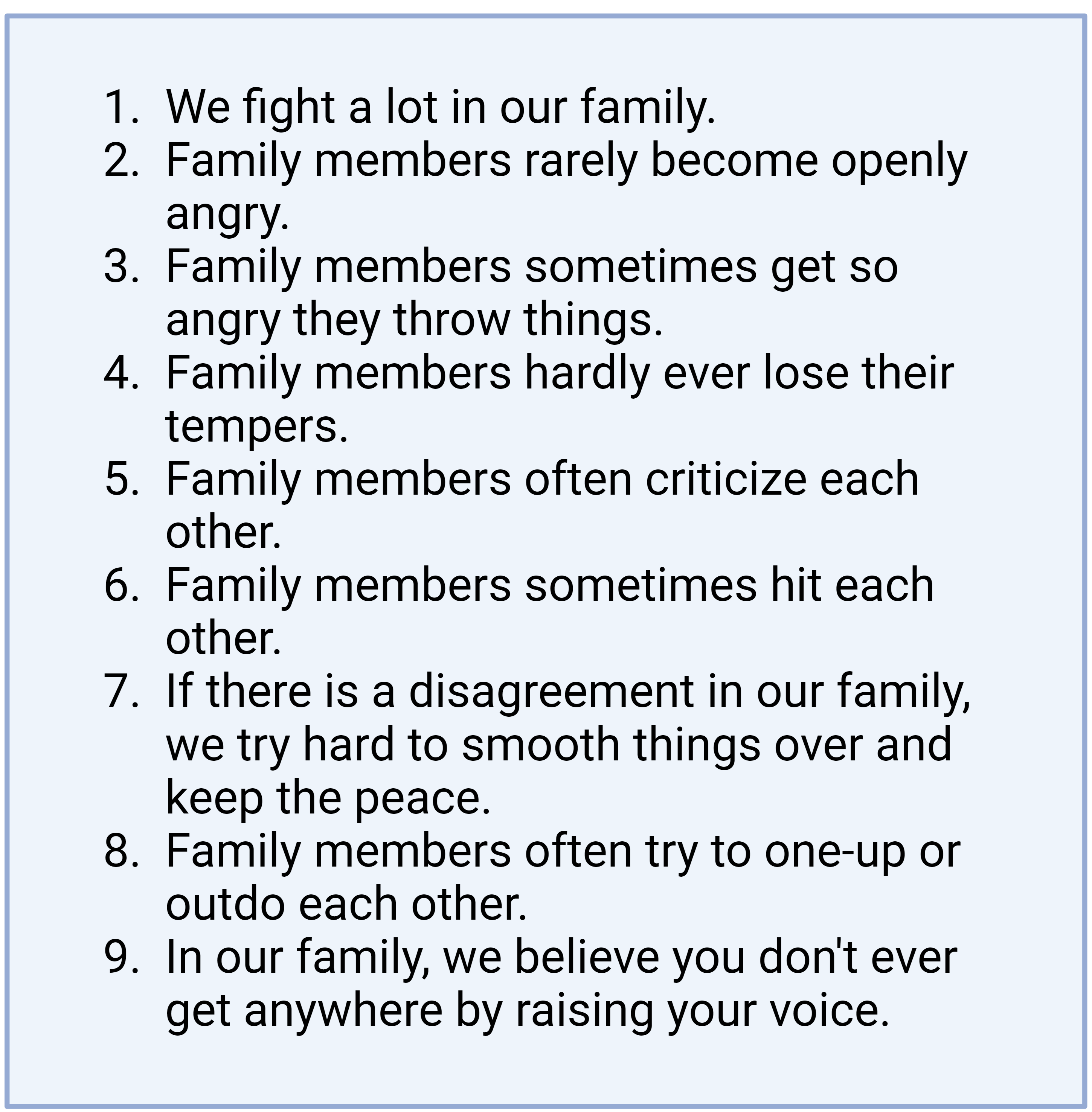
**

**Table S4. School Risk & Protective Factors**


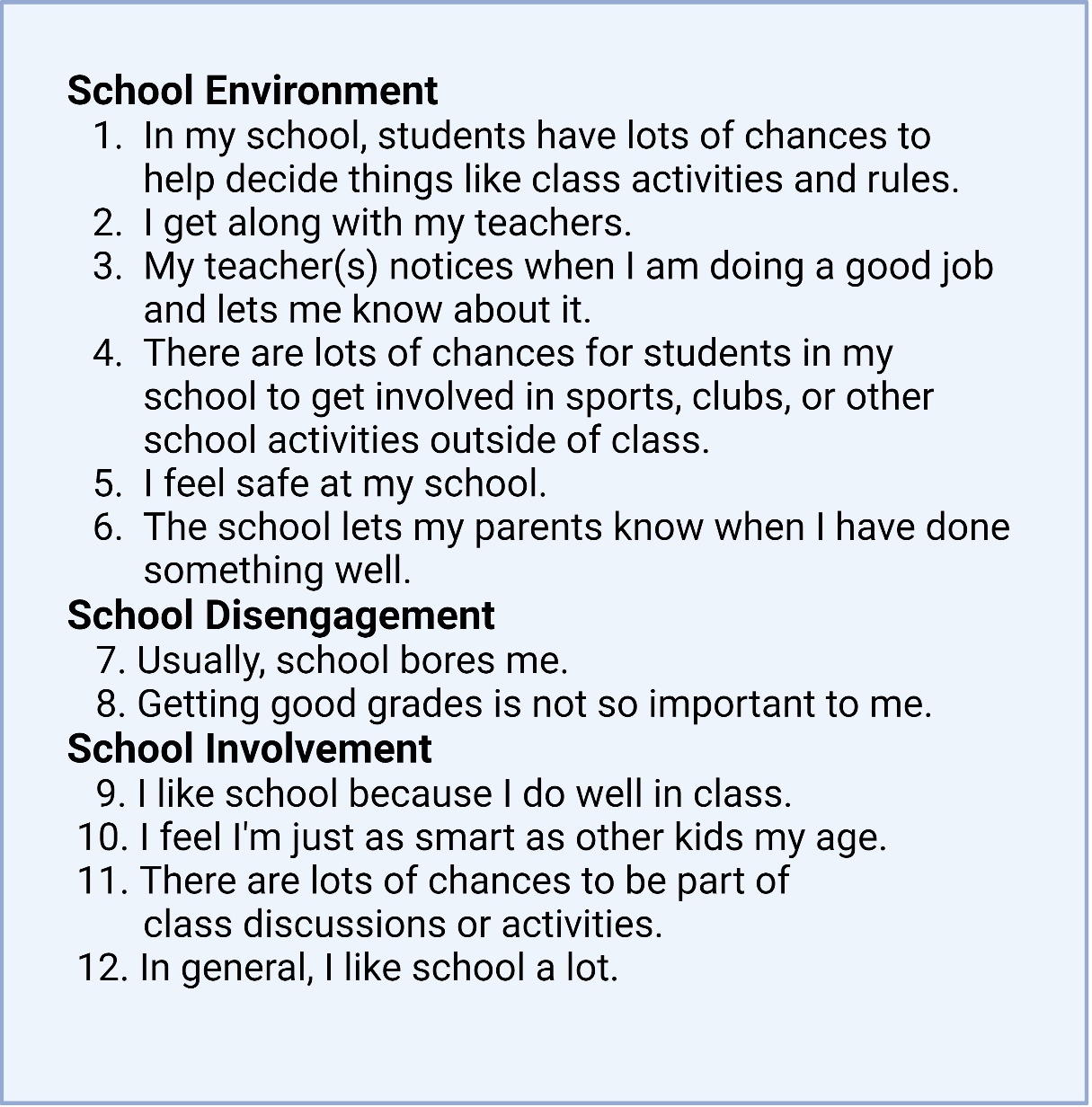


**Table S5. Prosocial Behavior questionnaire**


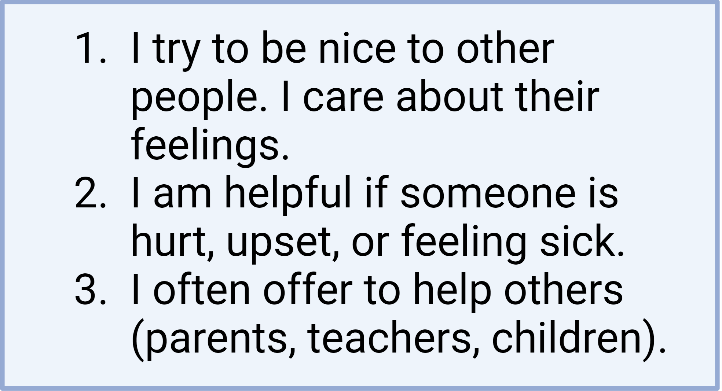


**Table S6. Peer Behavior Profile**


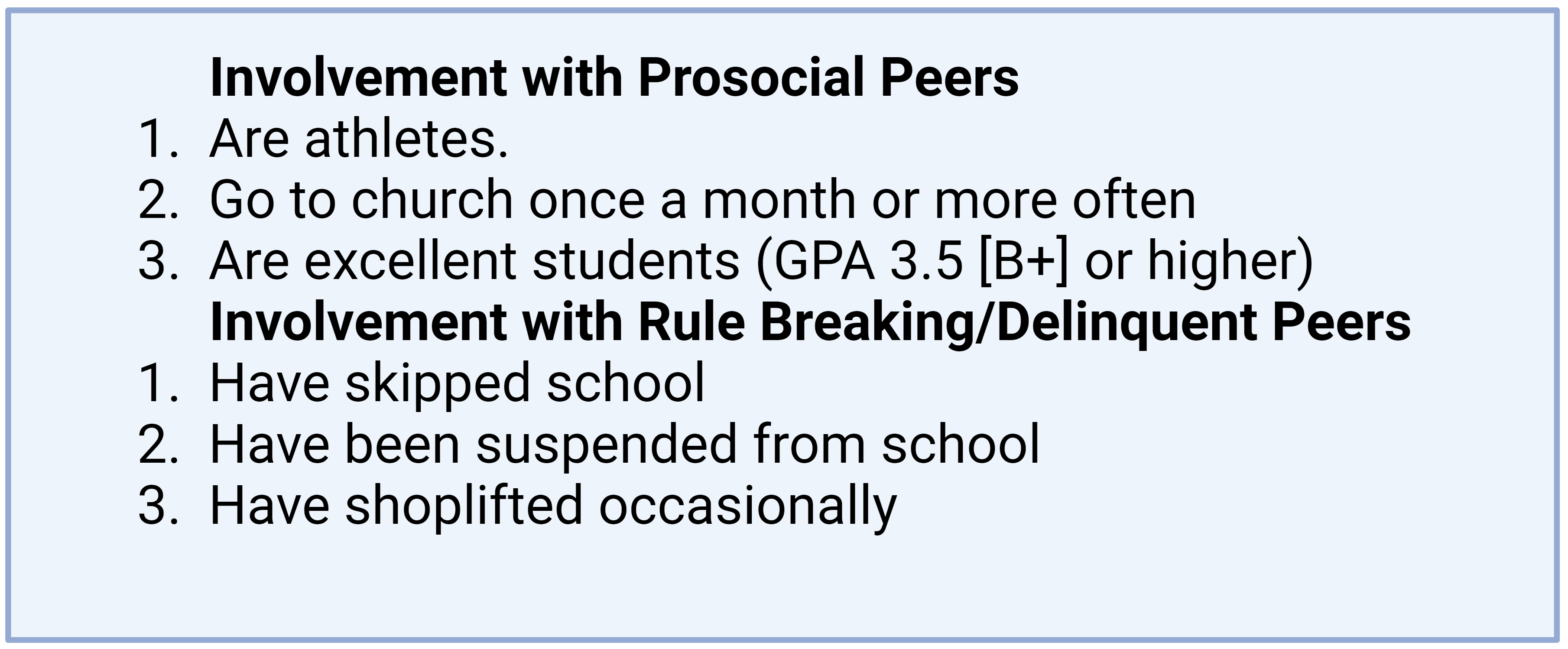


**Table S7. The Children’s Report of Parental Behavior Inventory**

**
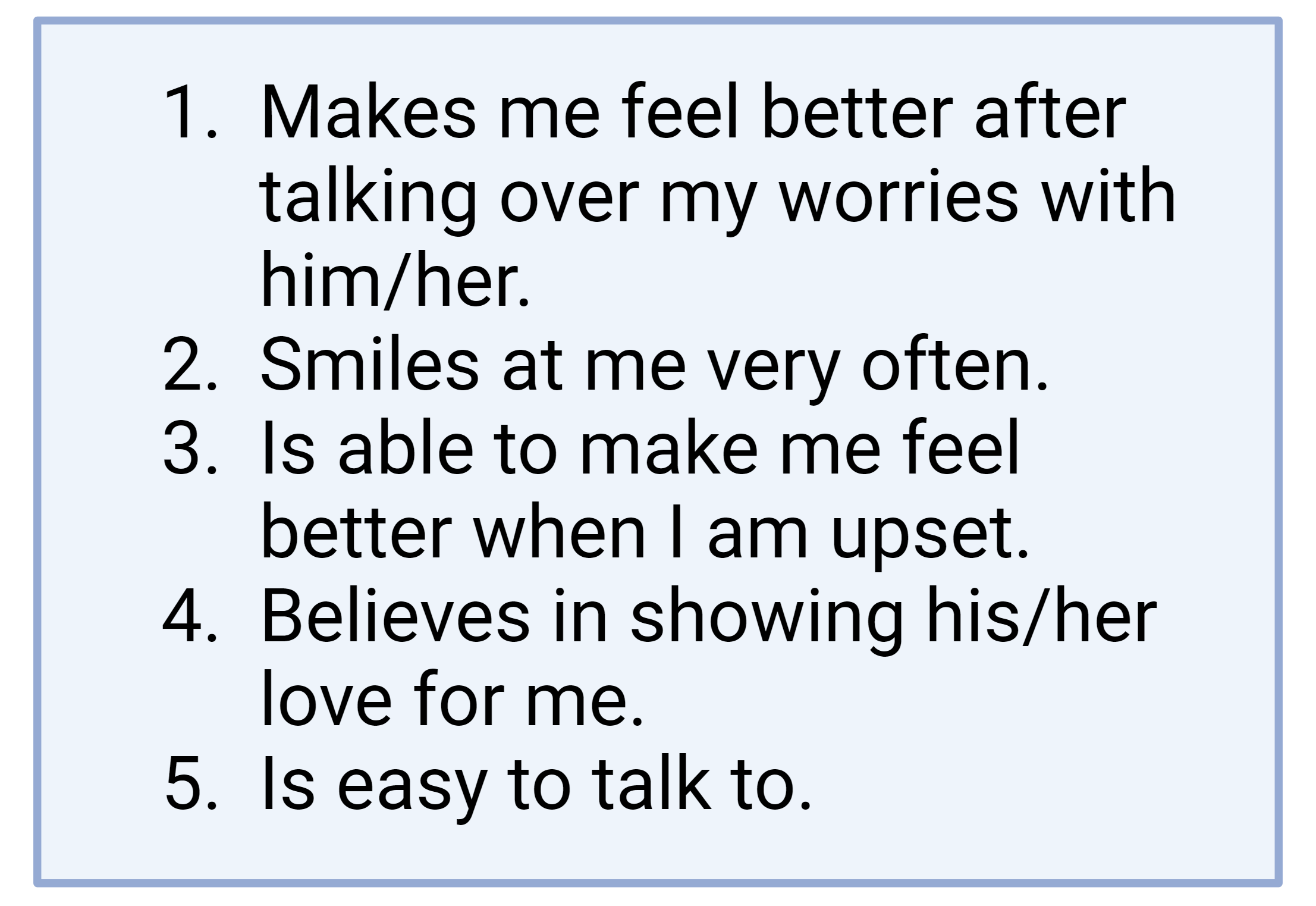
**

| **Table S8.** Missing data for variables including in the models | | |  |
| --- | --- | --- | --- |
|  | Number Missing | Overall (%) |  |
| Sex | 0 | 0.00 |  |
| Site | 0 | 0.00 |  |
| Age | 0 | 0.00 |  |
| ADHD diagnosis | 1879 | 6.03 |  |
| Household income | 511 | 1.63 |  |
| Marital status at baseline | 0 | 0.00 |  |
| Household education | 571 | 1.8 |  |
| Neighborhood crime at baseline | 108 | 0.05 |  |
| Family Conflict Scale | 83 | 0.02 |  |
| Parental Monitoring Scale | 72 | 0.02 |  |
| SRPF school involvement | 86 | 0.02 |  |
| SRPF school environment | 85 | 0.02 |  |
| SRPF school disengagement | 85 | 0.02 |  |
| Youth Prosocial Behaviors | 70 | 0.02 |  |
| PBP Prosocial Peer | 310 | 0.9 |  |
| PBP Rule Breaking Peer | 735 | 2.3 |  |
| Child’s Report - Parental Behavior Inventory | 295 | 0.9 |  |
| VGAQ | 6823 | 21.9 |  |
| Note. VGAQ = Video Game Addiction Questionnaire, SRPF = School Risk and Protective Factors, PBP = Prosocial Behavior Profile,  CRPBI = Child Report of Parental Behavior Inventory | | |  |
|  |  |  |  |


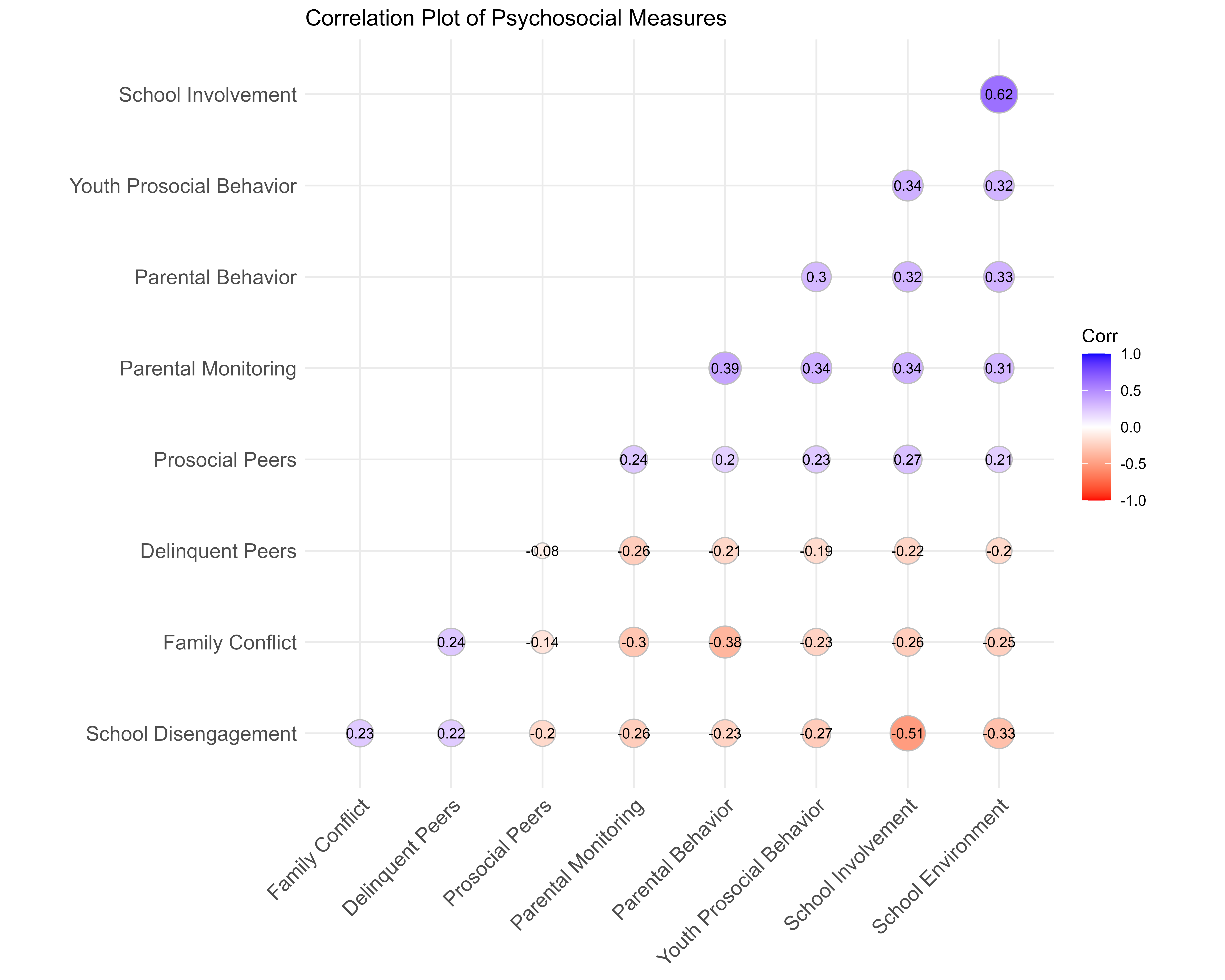


**Supplemental Figure 1.** Correlation matrix of the mediators included in the parallel mediation model.

| **Table S9.** Participant characteristics by mediator data completeness | | | |
| --- | --- | --- | --- |
|  | **No missing mediator data (n=9604)** | **Missing mediator data (n=1918)** | **p-value** |
| **Age in years (mean(SD))** | 9.96 (0.63) | 9.93 (0.61) | 0.063 |
| **Sex** |  |  | 0.048 |
| Male | 4987 (51.9) | 1044 (54.4) |  |
| Female | 4617 (48.1) | 874 (45.6) |  |
| **Household income** |  |  | <0.001 |
| < 25k | 1104 (11.5) | 434 (22.6) |  |
| 25k to 50k | 1218 (12.7) | 310 (16.2) |  |
| 50k to 75k | 1235 (12.9) | 215 (11.2) |  |
| 75k to 100k | 1363 (14.2) | 182 (9.5) |  |
| 100k to 200k | 2895 (30.1) | 367 (19.1) |  |
| > 200k | 1094 (11.4) | 141 (7.4) |  |
| Decline to answer | 356 (3.7) | 130 (6.8) |  |
| Do not know | 339 (3.5) | 138 (7.2) |  |
| **Household education** |  |  | <0.001 |
| < HS Diploma | 367 (3.8) | 187 (9.8) |  |
| HS Diploma/GED | 775 (8.1) | 297 (15.5) |  |
| Some College | 2338 (24.4) | 607 (31.7) |  |
| Bachelor's degree | 2581 (26.9) | 381 (19.9) |  |
| Post Graduate Degree | 3534 (36.8) | 442 (23.1) |  |
| **Marital status of parent/guardian** |  |  | <0.001 |
| Married | 6808 (70.9) | 1004 (52.4) |  |
| Widowed | 81 (0.8) | 14 (0.7) |  |
| Divorced | 847 (8.8) | 196 (10.2) |  |
| Separated | 331 (3.4) | 111 (5.8) |  |
| Never married | 1007 (10.5) | 378 (19.7) |  |
| Living with partner | 478 (5.0) | 184 (9.6) |  |
| **ADHD Present** |  |  | <0.001 |
| Yes | 649 (6.8) | 186 (9.7) |  |
| No | 8954 (93.2) | 1732 (90.3) |  |
| **Neighborhood Safety Score (mean(SD))** | 3.94 (0.94) | 3.69 (1.09) | <0.001 |
| **Note.** Values are n (%) unless otherwise indicated. Higher neighborhood safety scores reflect safer neighborhoods. | | | |

| **Table S10.** Comparison of model fit indices | | |
| --- | --- | --- |
|  | **Estimate** | ***p*** |
| **Model Fit Indices** | |  |
| Chi-square test | 1398.41 | <0.001* |
| RMSEA | 0.047 | 0.977** |
| CFI | 0.946 |  |
| TLI | 0.850 |  |
| SRMR | 0.073 |  |
| *DOF = 81. Scaling Correction = 1.2 | | |
| **Probability RMSEA <= 0.05 | | |

| **Table S11.** Individual path effects for the parallel mediation model | | |  |  |
| --- | --- | --- | --- | --- |
|  | **VGAQ at year 4** |  | **ADHD Diagnosis at year 2** |  |
|  | **β (SE)** | ***p*** | **β (SE)** | ***p*** |
| **Mediator at Year 3** |  |  |  |  |
| Parental Monitoring | -0.27 (0.2) | 0.19 | **-0.12 (0.02)** | **<0.001** |
| Family Conflict | **1.35 (0.5)** | **0.003** | **0.04 (0.01)** | **<0.001** |
| SRPF - School Disengagement | 0.19 (0.14) | 0.16 | **0.13 (0.04)** | **<0.001** |
| SRPF - School Involvement | **-0.61 (0.22)** | **0.005** | **-0.09 (0.03)** | **<0.001** |
| SRPF - School Environment | 0.16 (0.23) | 0.49 | **-0.06 (0.02)** | **0.011** |
| Prosocial Behavior Scale | -0.46 (0.27) | 0.09 | **-0.07 (0.02)** | **<0.001** |
| PBP Prosocial Peer Involvement | **-0.41 (0.1)** | **<0.0001** | **-0.18 (0.04)** | **<0.001** |
| PBP Rule Breaking/Delinquent Peer Involvement | 0.13 (0.2) | 0.53 | **0.07 (0.03)** | **0.008** |
| Parental Behavior Inventory | 0.27 (0.26) | 0.31 | **-0.09 (0.02)** | **<0.001** |
| **Note.** Significant relationships are in bold. SRPF = School Risk & Protective Factors, PBP = Peer Behavior Profile | | | | |


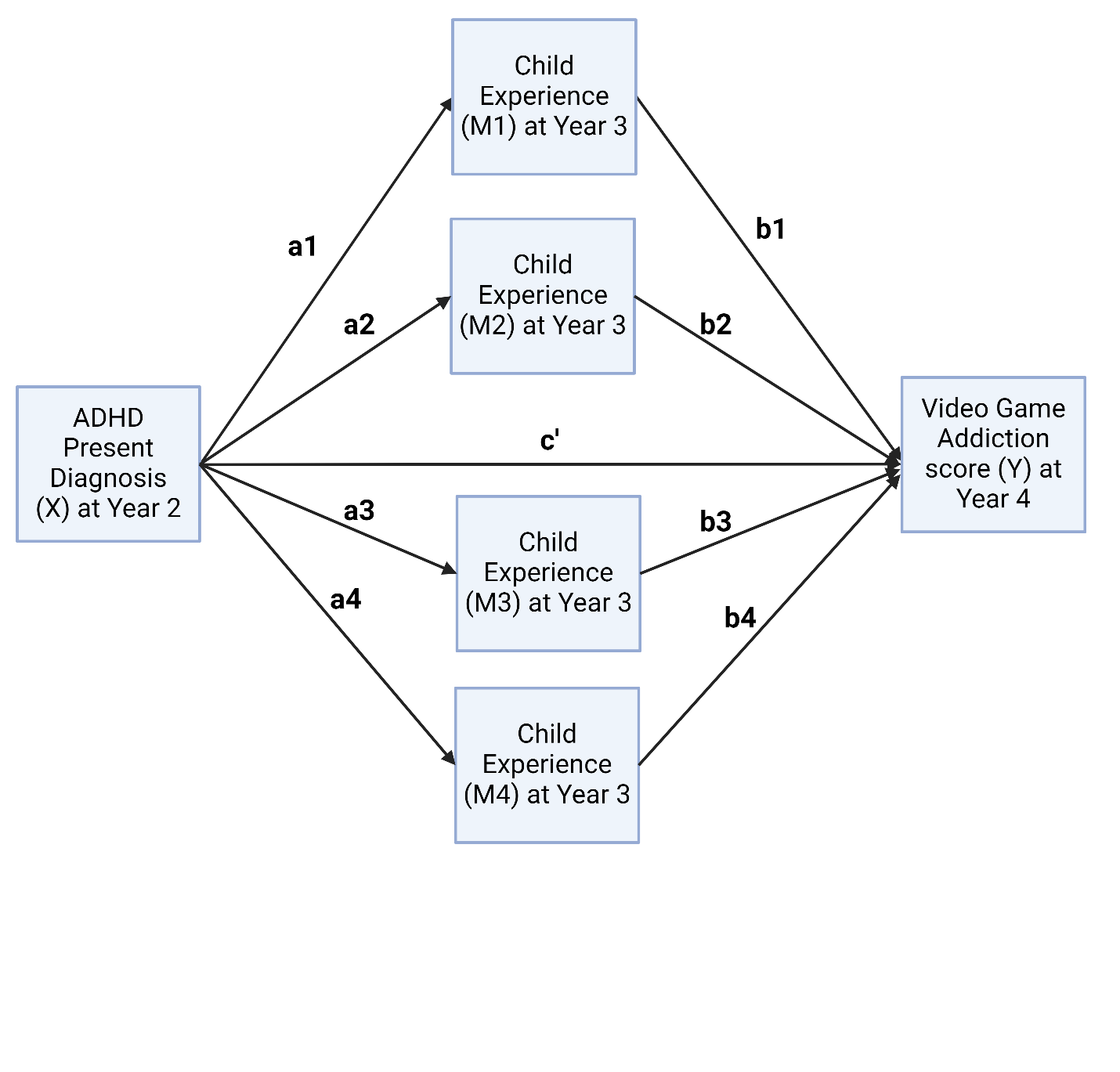


**Supplemental Figure 2** – Example of the temporal ordering of the parallel mediation model

**Supplementary Materials: Parallel mediation model details**

The temporal ordering of the exposure (X at year 2), mediators (M_1_, M_2_, M_3_…, M_n_ at year 3), and outcome (Y at year 4) eliminated the role of potential reverse causation in the longitudinal mediation analyses (i.e., child experiences at year 3 cannot cause an ADHD diagnosis at year 2 and a gaming addiction score at year 4 cannot cause child experiences at year 3) (59). The integration of multiple mediators also allows for a formal assessment of their relative importance (i.e., we can posit which mediators are the most influential contributors of the effect between ADHD diagnosis and VGAQ scores) (60). Additional details of the parallel mediation model can be found in the supplementary materials.

The parallel mediation model included a single regression for the outcome of VGAQ score and a separate regression for each mediator. The single regression for VGAQ score included simultaneous adjustment for 9 mediators.

An example of a 3-mediator model is shown below:

$M_{1}= i_{M1}+a_{1}X+ e_{M1}$ (1.1)

$M_{2}=i_{M2}+a_{2}X+e_{M2}$ (1.2)

$M_{3}=i_{M3}+a_{3}X+e_{M3}$ (1.3)

$Y=i_{y}+c^{'}X+b_{1}M_{1}+{b_{2}M_{2}+b_{3}M_{3}+ e}_{y}$ (1.4)

In the mediator models (1.1 through 1.3), a_1_, a_2_, and a_3_ represent the beta coefficients associated with a diagnosis of ADHD. A participant with a diagnosis of ADHD (1=Yes) consequently has an increase in M of the corresponding amount. The outcomes model (1.4) includes all the mediators (M_1_, M_2_, M_3_…, M_9_) that are a part of the analysis and are held constant rather than viewed in isolation. For example, a one unit increase in the beta coefficient of M_1_ is associated with a corresponding change in Y, after holding X, M_2_, M_3_, and other mediators and covariates in the model constant. The change in X can be interpreted as the estimated mean difference between the two groups (i.e., those with and without an ADHD diagnosis). The specific indirect effect of each mediator is calculated by multiplying the effects of path X_i_ to M_i_ by the effects of M_i_ to Y_i_. Consequently, the results can be interpreted as the change in Y after controlling for all other mediators in the model. The total indirect effect is calculated by summing the individual indirect effects for each mediator. Like in traditional mediation, the total effect is the sum of the direct and indirect effects.
